# Effect of Spicatoside a on Anti-Osteosarcoma MG63 Cells through Reactive Oxygen Species Generation and the Inhibition of the PI3K-AKT-mTOR Pathway

**DOI:** 10.3390/antiox13101162

**Published:** 2024-09-25

**Authors:** Hyung-Mun Yun, Soo Hyun Kim, Yoon-Ju Kwon, Kyung-Ran Park

**Affiliations:** 1 Department of Oral and Maxillofacial Pathology, School of Dentistry, Kyung Hee University, Seoul 02447, Republic of Korea; yunhm@khu.ac.kr; 2 National Development Institute for Korean Medicine, Gyeongsan 38540, Republic of Korea; beluga81@nikom.or.kr (S.H.K.); mars005@nikom.or.kr (Y.-J.K.); 3 Korea Basic Science Institute (KBSI), Gwangju 61751, Republic of Korea

**Keywords:** Spicatoside A, ROS, osteosarcoma, necroptosis, autophagy, apoptosis, AKT

## Abstract

Osteosarcoma is a primary malignant tumor found in the bones of children and adolescents. Unfortunately, many patients do not respond well to treatment and succumb to the illness. Therefore, it is necessary to discover novel bioactive compounds to overcome therapeutic limitations. *Liriope platyphylla* Wang et Tang is a well-known herb used in oriental medicine. Studies have shown that metabolic diseases can be clinically treated using the roots of *L. platyphylla*. Recent studies have demonstrated the anticarcinoma potential of root extracts; however, the exact mechanism remains unclear. The aim of this study was to examine the anti-osteosarcoma activity of a single compound extracted from the dried roots of *L. platyphylla*. We purified Spicatoside A (SpiA) from the dried roots of *L. platyphylla*. SpiA significantly inhibited the proliferation of human osteosarcoma MG63 cells in a dose- and time-dependent manner. SpiA also regulated the expression of various downstream proteins that mediate apoptosis (PARP, Bcl-2, and Bax), cell growth (cyclin D1, Cdk4, and Cdk6), angiogenesis (VEGF), and metastasis (MMP13). The Proteome Profiler Human Phospho-Kinase Array Kit showed that the AKT signaling protein was a target of SpiA in osteosarcoma cells. We also found that SpiA suppressed the constitutive activation of the PI3K-AKT-mTOR-p70S6K1 signaling pathway. We further validated the effects of SpiA on the AKT signaling pathway. SpiA induced autophagosome formation and suppressed necroptosis (a form of programmed cell death). SpiA increased the generation of reactive oxygen species (ROS) and led to the loss of mitochondrial membrane potential. N-acetylcysteine (NAC)-induced inhibition of ROS generation reduced SpiA-induced AKT inhibition, apoptotic cell death, and anti-metastatic effects by suppressing cell migration and invasion. Overall, these results highlight the anti-osteosarcoma effect of SpiA by inhibiting the AKT signaling pathway through ROS generation, suggesting that SpiA may be a promising compound for the treatment of human osteosarcoma.

## 1. Introduction

Osteosarcoma is the most common malignant bone tumor in children and adolescents and originates from osteoblasts of mesenchymal origin [1,2,3]. It is microscopically characterized by an abnormally formed osteoid, known as a tumor osteoid [4]. In cases where the osteosarcoma has not metastasized to other body parts, the survival rate is 70%. However, if the cancer has already progressed to the lungs, bones, or other tissues at the time of diagnosis, the survival rate decreases to approximately 30% [5]. Approximately 20% of patients with osteosarcoma have detectable metastases at the time of diagnosis [6]. Despite research efforts, there has been little improvement in the five-year survival rate [7]. Although many different therapies have been proposed for osteosarcoma, chemotherapy and surgery are the most advanced clinical approaches [1]. Chemotherapy is a crucial adjuvant therapy for osteosarcoma [8]. Although patients with osteosarcoma now have a progressively higher survival rate due to adjuvant and neoadjuvant chemotherapy, 20–40% of patients still develop distant metastases or local recurrences [9]. Therefore, new chemotherapeutic agents for osteosarcoma must be identified based on their biological effects.

AKT serine–threonine kinase (AKT, also known as protein kinase B) and oxidative stress regulate various cellular processes in cancer cells, and several natural compounds exhibit anti-cancer properties by modulating these processes [10]. AKT regulates numerous cellular functions through its downstream proteins [11]. The AKT signaling pathway plays a crucial role in a wide range of physiological and pathological processes, especially in most common human cancers [12]. In osteosarcoma, aberrant AKT signaling influences pathological processes such as proliferation, apoptosis, migration, invasion, metastasis, autophagy, angiogenesis, and chemoresistance [13]. Oxidative stress is characterized by elevated levels of reactive oxygen species (ROS) within cells, which serve as crucial regulators of signaling pathways [14]. A growing variety of treatment approaches have been developed that elevate ROS levels to induce oxidative stress [14,15,16,17,18,19]. ROS inhibit the AKT signaling pathway in various cancer cells and exert anti-cancer effects [20,21,22,23]. ROS also inactivate AKT by direct oxidization and dephosphorylation [24]. Thus, to improve the survival rate of patients with osteosarcoma, the AKT signaling pathway has garnered significant attention and has been the focus of various small-molecule medications.

The perennial plant *Liriope platyphylla* Wang et Tang has been used traditionally to produce herbal medicines in several Asian countries. The roots of *L. platyphylla* have been widely used for various purposes, including tea production and medicines [25]. There is significant potential for the development of *L. platyphylla* as a treatment for chronic human diseases such as inflammation, diabetes, neurodegenerative disorders, obesity, and atopic dermatitis [25]. Recent studies have demonstrated that compounds derived from the roots of *L. platyphylla* exhibit anti-cancer properties [26]. (−)-Liriopein B, derived from *L. platyphylla* roots, suppresses cancer progression in human breast cancer cells by inhibiting various kinases, including EGFR, FGFR1, PI3K, RTK5, Src, Flt, Tie2, and Abl [27].

Steroidal saponins, including Spicatoside A (SpiA) from *L. platyphylla* roots, have been shown to increase apoptosis and reduce the proliferation of human non-small cell lung and colorectal cancer cells [28,29]. In addition to its inhibitory effects on cell growth in various carcinoma cells, SpiA has a range of other benefits, including anti-inflammatory properties, anti-asthma effects, inhibition of osteoclastogenesis, promotion of neurite outgrowth, and enhancement of memory consolidation [30]. Although the effects of *L. platyphylla* root extract on several carcinomas have been studied, the exact mechanism is not yet fully understood. In particular, its anti-cancer effects on sarcomas remain unexplored. Therefore, it is worthwhile to investigate the potential activities and biological mechanisms of *L. platyphylla* root-derived compounds against osteosarcoma.

In the present study, we isolated SpiA with a purity greater than 99% from the dried roots of *L. platyphylla* and investigated its anti-osteosarcoma properties using human osteosarcoma MG63 cells.

## 2. Materials and Methods

### 2.1. Plant Material and General Procedures

The roots of *L. platyphylla* Wang et Tang were purchased from Omniherb, Daegu, Republic of Korea. A voucher specimen (P392) was deposited in the Natural Products Bank of the National Institute for Korean Medicine Development (NIKOM). For compound extraction, 15 kg dried *L. platyphylla* roots was used in this study. Nuclear magnetic resonance (NMR) was conducted on a JEOL ECX-500 spectrometer (JEOL Ltd., Tokyo, Japan) operating at 500 MHz for ^1^H and 125 MHz for ^13^C. High-performance liquid chromatography (HPLC) was performed using Agilent 1200 series (Agilent Technologies, Santa Clara, CA, USA) with the parameters listed in Table 1. Column chromatography was performed using a Diaion HP-20 (Mitsubishi Chem. Co., Tokyo, Japan) and ODS-A (YMC Co., Ltd., Kyoto, Japan).

### 2.2. Extraction and Isolation

The dried roots of *L. platyphylla* (15 kg) were incubated in 80% MeOH for 2 days (3 × 90 L). The 272.8 g of MeOH extract was dried and suspended in 6.9 L of deionized water, and ethyl acetate and n-butanol were used to partition the solvent. Eight fractions (LPB 1–LPB 8) were obtained by eluting the n-butanol-soluble fraction (99.39 g) with a gradient of H_2_O-MeOH (100:0 to 0:100, *v*/*v*) using Diaion HP-20 column chromatography. To extract the active substance (820 mg), fractions LPB 6 and LPB 7 (3.24 g) were subjected to reverse-phase (ODS-A) column chromatography and eluted using a gradient of MeOH-H2O (50:50 to 0:100, *v*/*v*). The solution was then recrystallized in MeOH-H_2_O to produce high-purity SpiA (122 mg of white powder).

### 2.3. SpiA Stock Solution

A 1000× SpiA stock solution was prepared by dissolving the SpiA powder in 100% dimethyl sulfoxide (DMSO) (Sigma-Aldrich, St. Louis, MO, USA). During experiments, 0.1% DMSO was added as a vehicle control.

### 2.4. Cell Culture

The American Type Culture Collection (ATCC, Manassas, VA, USA) provided human osteosarcoma MG63 cells (#CRL-1427) isolated from the bones of a Caucasian male patient who was 14 years old. The cells were cultured in an incubator at 37 °C with an atmosphere of 95% air and 5% CO_2_. The culture medium used was Dulbecco’s modified Eagle medium (WELGEM, Inc., Seoul, Republic of Korea) with 10% fetal bovine serum (Thermo Fisher Scientific, Waltham, MA, USA) and 1× Gibco^®^ antibiotic-antimycotic (Thermo Fisher Scientific), as previously described [31].

### 2.5. 3-[4,5-Dimethylthiazol-2-yl]-2,5-diphenyltetrazolium Bromide (MTT) Assay

The MTT assay (Sigma-Aldrich) was used to evaluate cell viability, as previously described [31]. Cells were incubated with 20 µL of MTT solution (5 mg/mL in PBS). The dark purple crystalline formazan was dissolved in 100% DMSO and analyzed spectrophotometrically at a wavelength of 540 nm using a Multiskan GO Microplate Spectrophotometer (Thermo Fisher Scientific).

### 2.6. BrdU Cell Proliferation Assay

A BrdU Cell Proliferation ELISA Kit (colorimetric) (Abcam, Cambridge, UK) was used to assess proliferation and apoptosis by quantifying DNA replication, following the manufacturer’s protocol.

### 2.7. Western Blotting

Western blotting was performed as described previously [32]. The following antibodies were used: Bax (1:1000, #2772), Bcl-2 (1:1000, #15071), PARP (1:1000, #9542), LC3A/B (1:1000, #12741), Beclin1 (1:1000, #3495), p62 (1:1000, #5114), RIP (1:1000, #3493), p-RIP (1:1000, #65746), RIP3 (1:1000, #13526), pRIP3 (1:1000, #93654), MLKL (1:1000, #14993), p-MLKL (1:1000, #91689), p-PI3K (1:1000, #4228), PI3K (1:1000, #4257), p-mTOR (1:1000, #2974), mTOR (1:1000, #2983), AKT (1:1000, #4691), p-AKT (1:1000, #4060), p-p70S6K (1:1000, #9204), and p70S6K (1:1,000, #2708) obtained from Cell Signaling Technology (Beverly, MA, USA); MMP13 (1:1000, NBP1-45723) obtained from Novus Biologicals (Centennial, CO, USA); and CyclinD1 (1:1000, #sc-20044), Cdk4 (1:1000, #sc-23896), Cdk6 (1:1000, #sc-7961), and β-actin (C4, 1:1000, #sc-47778) obtained from Santa Cruz Biotechnology (Dallas, TX, USA).

### 2.8. Proteome Profiler Human Phospho-Kinase Array Kit

The Proteome Profiler Human Phospho-Kinase Array Kit (#ARY003C, R&D Systems, Minneapolis, MN, USA) was used to detect the phosphorylation of multiple human kinases simultaneously. Antibodies against human phosphokinases were spotted onto the membranes in duplicate. The subsequent processes were performed in compliance with the supplier’s protocol. Signals were detected using a ChemiDoc Imaging System (Bio-Rad Laboratories, Inc., Hercules, CA, USA).

### 2.9. Autophagosome Formation Assay

DAPGreen (Dojindo, Tokyo, Japan) was used to detect autophagy as described previously [31]. Images were captured using an Olympus IX73 inverted microscope (Olympus Corporation, Tokyo, Japan) and an intravital multi-photon microscope system (KJ316; Leica Microsystems, Wetzlar, Germany) at the Korea Basic Science Institute (KBSI, Gwangju, Republic of Korea).

### 2.10. ROS and Mitochondrial Membrane Potential

CellROX™ Green reagent (Invitrogen, Carlsbad, CA, USA), MitoTracker™ Red CMXRos (Invitrogen), and Rhodamine 123 (Invitrogen) were used to detect ROS levels and mitochondrial membrane potential as described previously [31].

### 2.11. Terminal Deoxynucleotidyl Transferase-Mediated FITC–dUDP Nick-End Labeling (TUNEL) Assay

TUNEL assays were performed to detect apoptosis by quantifying DNA fragmentation using an in situ Cell Death Detection Kit (Roche Diagnostics, Mannheim, Germany) in compliance with the manufacturer’s protocol, as previously described [33].

### 2.12. Cell Migration Assay

A wound-healing assay was performed to measure cell migration, as previously described [34]. Images were captured using a light microscope (Olympus Corporation).

### 2.13. Cell Invasion Assay

Cell invasion was assessed using a Boyden chamber with membranes coated with the Matrigel solution (Corning Life Sciences, Tewksbury, MA, USA) as previously described [35]. Images were captured using a light microscope (Olympus Corporation).

### 2.14. Statistical Analysis

Statistical significance (*p* < 0.05) was assessed using one-way analysis of variance, followed by Dunnett’s post hoc test using GraphPad Prism version 5 (GraphPad Prism, Inc., San Diego, CA, USA). Data are presented as mean ± standard deviation (SD).

## 3. Results

### 3.1. Isolation and Characterization of SpiA from L. platyphylla

**Figure 1:**
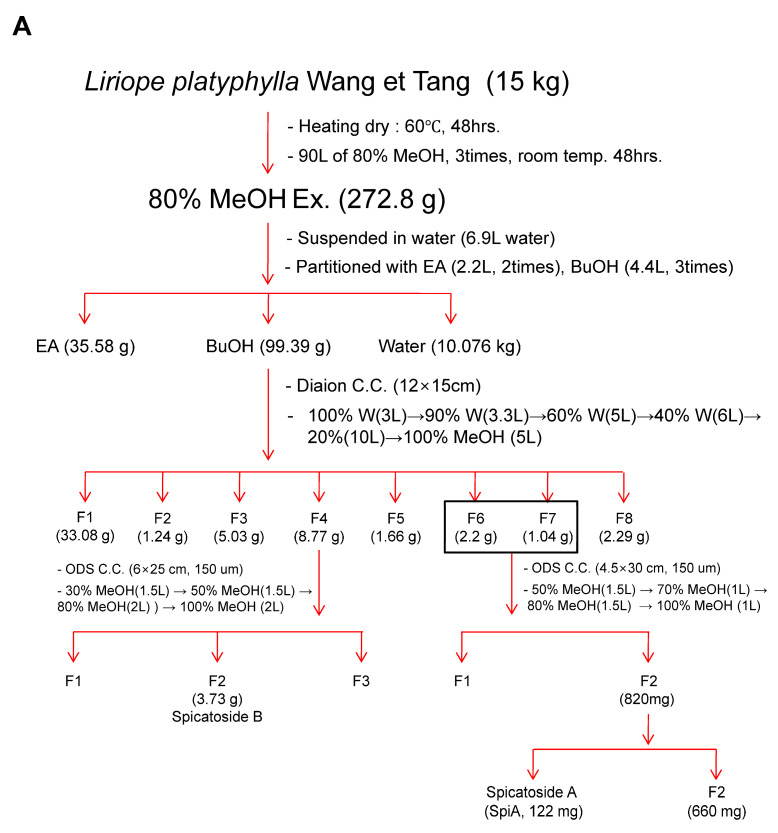

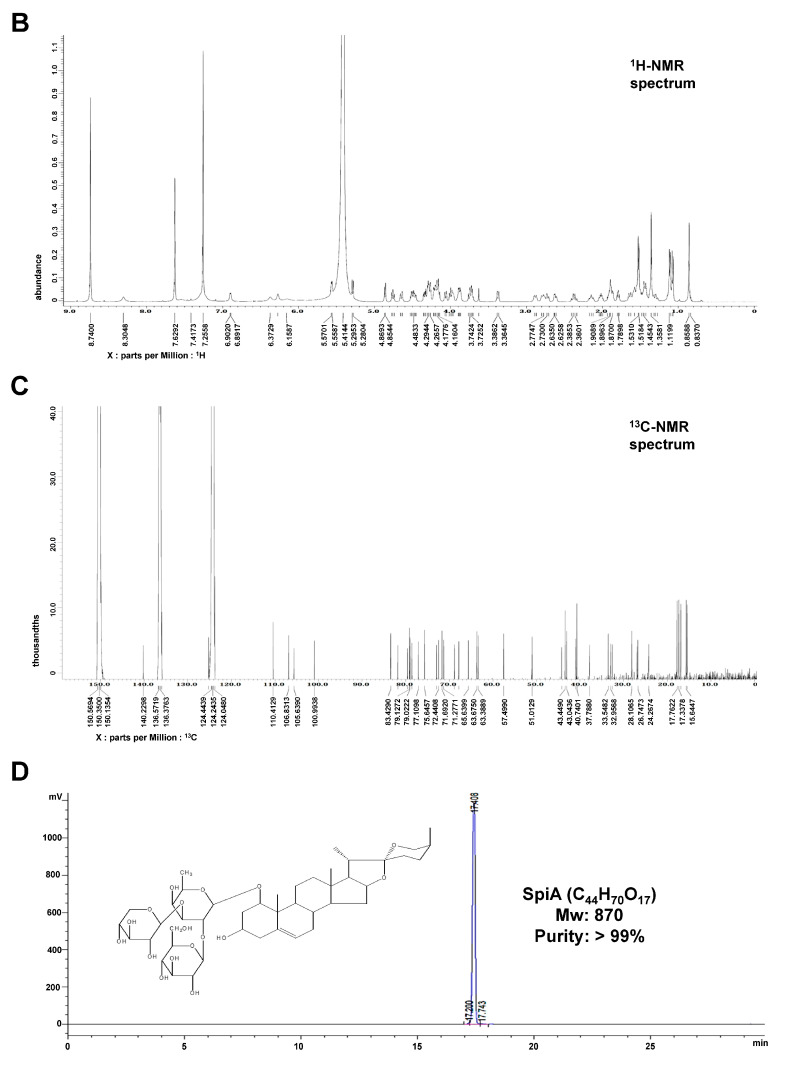
Isolation and characterization of Spicatoside A (SpiA) from *Liriope platyphylla* roots. (**A**) The strategy for extracting SpiA. (**B**,**C**) ^1^H NMR (500 MHz, Pyridine-*d_5_*) (**B**) and ^13^C-NMR (125 MHz, Pyridine-*d_5_*) (**C**) spectra of SpiA. (**D**) HPLC evaluation and chemical structure of isolated SpiA.

SpiA was isolated from 15 kg of *L. platyphylla* roots using the process described in Figure 1A. The ^1^H-NMR (500 MHz, Pyridine-*d*_5_) spectrum displayed peaks at δ 5.57 (1H, d, *J* = 5.7 Hz, H-6), 5.29 (1H, d, *J* = 7.4 Hz, H-anomeric), 4.86 (1H, d, *J* = 7.6 Hz, H-anomeric), 1.53 (3H, d, *J* = 6.3 Hz, fuc-6), 1.35 (3H, s, H-19), 1.11 (3H, d, *J* = 6.8 Hz, H-21), 1.08 (3H, *J* = 7.4 Hz, H-27), and 0.85 (3H, s, H-18) (Figure 1B). The ^13^C-NMR (125 MHz, Pyridine-*d*_5_) spectrum displayed peaks at δ 140.2 (C-5), 125.0 (C-6), 110.4 (C-22), 106.8 (xyl-1), 105.6 (glc-1), 100.9 (fuc-1), 83.4 (fuc-3), 83.4 (C-1), 81.8 (C-16), 79.5 (fuc-2), 79.1 (glc-3), 79.0 (glc-5), 78.5 (xyl-3), 77.1 (glc-2), 75.6 (xyl-2), 72.9 (fuc-4), 72.4 (glc-4), 71.6 (fuc-5), 71.2 (xyl-4), 68.8 (C-3), 67.8 (xyl-5), 65.6 (C-26), 63.6 (C-17), 63.3 (glc-6), 57.4 (C-14), 51.0 (C-9), 44.2 (C-4), 43.4 (C-10), 43.0 (C-20), 40.9 (C-12), 40.7 (C-13), 37.7 (C-2), 33.5 (C-8), 32.9 (C-7), 32.6 (C-15), 28.1 (C-25), 26.9 (C-23), 26.7 (C-24), 24.2 (C-11), 17.7 (fuc-6), 17.3 (C-18), 16.8 (C-27), 15.6 (C-21), and 15.4 (C-19) (Figure 1C). The SpiA extracted was a white powder with a purity above 99% and chemical formula C_44_H_70_O_178_. Figure 1D shows the chemical structure of SpiA (inset) and HPLC results.

### 3.2. SpiA Suppresses Proliferation and Induces Apoptotic Cell Death in Human MG63 Cells

**Figure 2:**
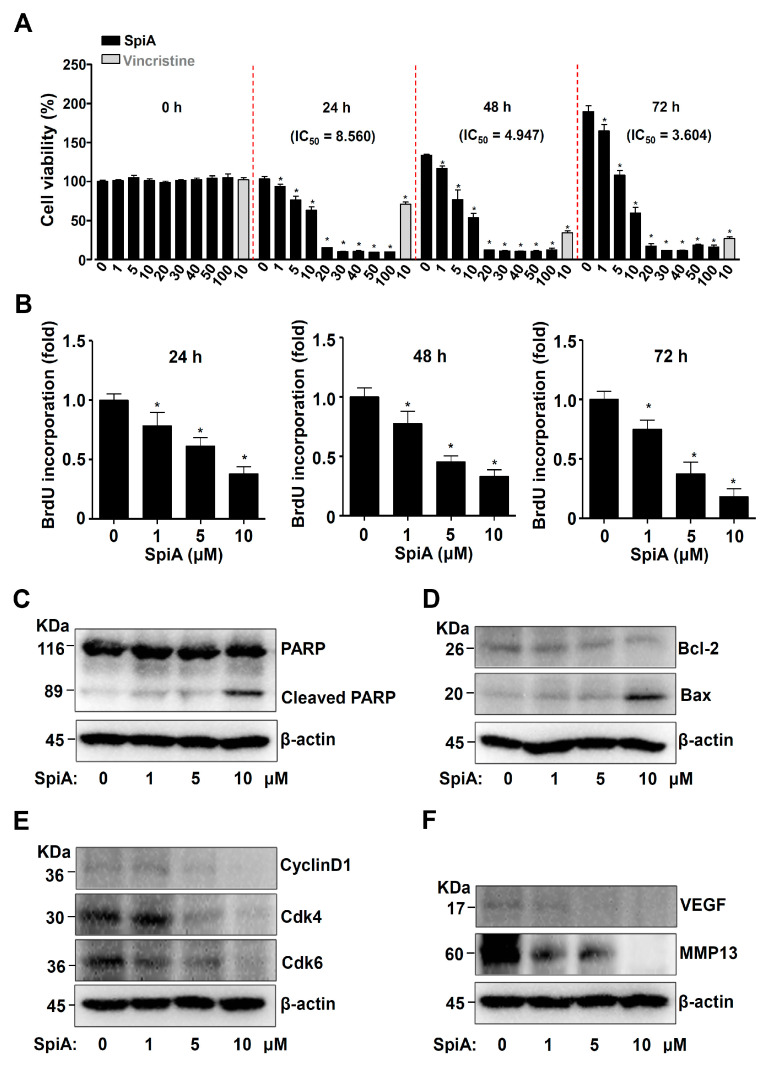
Anti-tumor effects of SpiA on cell proliferation and apoptosis in human MG63 cells. (**A**) MTT assay after SpiA or Vincristine treatment with the indicated doses for 3 days. The red dotted line indicates the boundary marks for 0, 24, 48, and 72 h. (**B**) BrdU cell proliferation assay after SpiA with the indicated doses for 3 days in cells. (**C**–**F**). Western blotting was performed after SpiA treatment with the indicated doses for 24 h. Western blotting of PARP, cleaved PARP (**C**), Bcl-2, Bax (**D**), cyclin D1, Cdk4/6 (**E**), VEGF, and MMP13 (**F**). The total levels of β-actin were used as a loading control for the samples. * indicates statistical significance at *p* < 0.05. Representative results of three independent experiments.

To assess the effects of the SpiA isolated from *L. platyphylla* Wang et Tang on proliferation, human osteosarcoma MG63 cells were treated with 1–100 μM SpiA for 3 days. Vincristine (10 μM), a chemotherapy agent, was used as a positive control. The results of the MTT assay indicated that SpiA significantly reduced proliferation in a dose- and time-dependent manner in human MG63 cells (Figure 2A). In the subsequent experiments, human MG63 cells were treated with 1 to 10 μM SpiA. Using BrdU cell proliferation assay, we then confirmed that the treatment of SpiA reduced BrdU incorporation, thereby indicating the effect of SpiA on cell proliferation (Figure 2B).

We next investigated the effects of SpiA on cell death. Firstly, we analyzed PARP cleavage, a hallmark of apoptotic cell death. The cleavage of PARP was increased following treatment with SpiA compared to that in untreated cells, as observed by Western blotting (Figure 2C). Secondly, SpiA treatment increased pro-apoptotic Bax protein levels and lowered anti-apoptotic Bcl-2 protein levels (Figure 2D). Thirdly, SpiA inhibited cyclin D1 and Cdk4/6, which are linked to cell division, as well as proteins associated with angiogenesis and metastasis, such as VEGF and MMP13 (Figure 2E,F). These findings suggested that SpiA mediates apoptotic cell death by regulating apoptotic, proliferative, angiogenic, and metastatic proteins in human MG63 cells.

### 3.3. SpiA Suppresses the PI3K-AKT-Mammalian Target of Rapamycin (mTOR)-p70S6K Pathway in Human MG63 Cells

**Figure 3:**
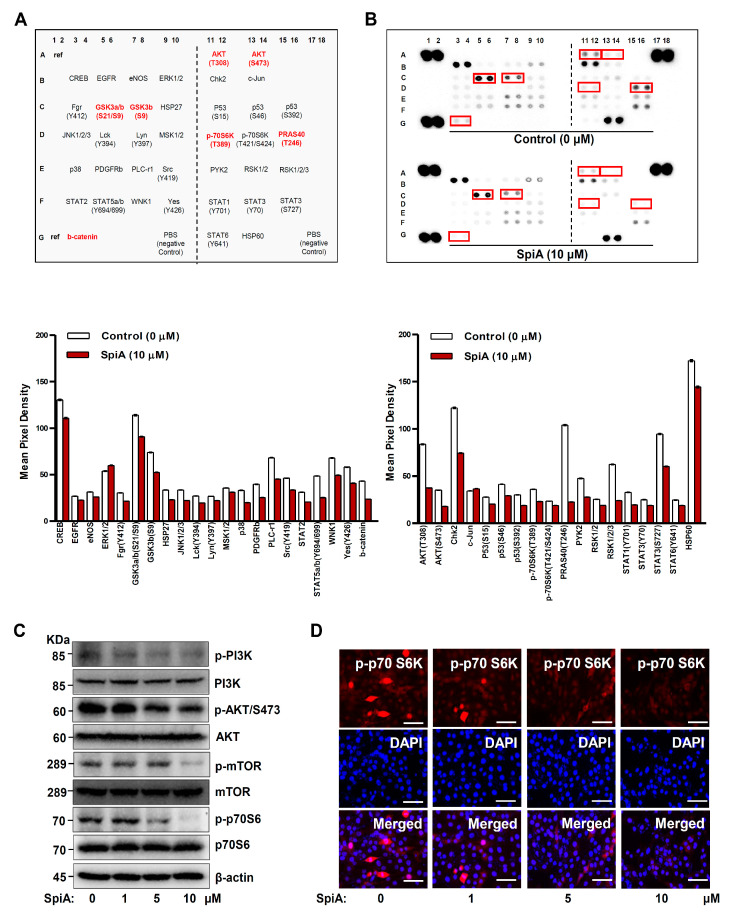
Anti-tumor effects of SpiA on AKT signaling in human MG63 cells. (**A**) Kinases in the Proteome Profiler Human Phospho-Kinase Array are shown in the table. (**B**) The phosphorylation profiles of 37 different kinases were analyzed after SpiA treatment for 24 h. Red rectangles indicate double spots with large differences. The density is illustrated in a bar graph. (**C**) Western blotting was performed after SpiA treatment with the indicated doses for 24 h. (**D**) Immunofluorescence assay to monitor phosphorylation levels of p70S6K (red) after SpiA treatment with the indicated doses for 24 h. DAPI staining (blue) indicates nuclei. Scale bar: 50 μm. Representative results of three independent experiments.

By screening for the phosphorylation of intracellular SpiA proteins, we investigated the signaling molecules involved in apoptotic cell death in human MG63 cells. The phosphorylation levels of proteins associated with the protein kinase B (AKT) pathway (AKT, 70-kDa ribosomal protein S6 kinase (p70S6K), 40 kDa proline-rich Akt substrate (PRAS40), GSK3β, and β-catenin) were decreased compared to those of the untreated cells, as determined by a human phospho-kinase array (Figure 3A,B). Western blotting confirmed a significant decrease in the phosphorylation of the major PI3K-AKT-mTOR-p70S6K signaling pathway, which was consistent with the screening results (Figure 3C). We also observed the phosphorylation of p70S6K using a fluorescence microscope. SpiA treatment decreased the division of human MG63 cells, as demonstrated by p-p70S6K- and DAPI-stained cells (Figure 3D); p70S6K is a mitogen-activated Ser/Thr protein kinase required for cell proliferation and division. These results suggested that the PI3K-AKT-mTOR-p70S6K pathway is a target of SpiA, producing its anti-tumor effects in human MG63 cells.

### 3.4. SpiA Induces Autophagy and Inhibits Necroptosis in Human MG63 Cells

**Figure 4:**
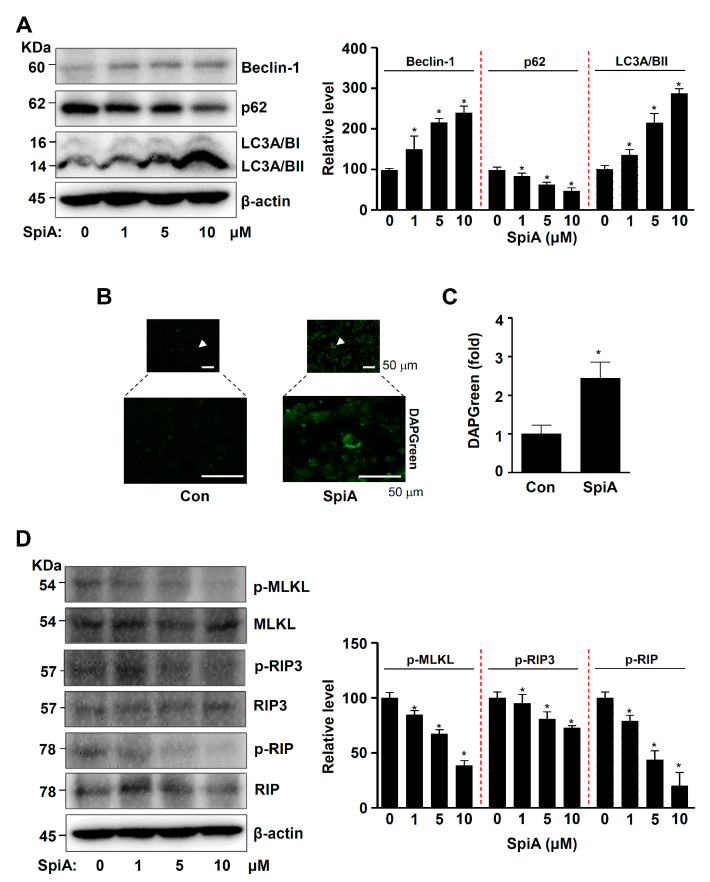
Anti-tumor effects of SpiA on autophagic and necroptotic processes in human MG63 cells. (**A**) After SpiA treatment with the indicated doses for 24 h, Western blotting was performed to assess autophagy. The relative level (%) normalized to β-actin is illustrated in the bar graph. The red dotted line indicates the boundary marks. (**B**,**C**) Immunofluorescence assay to monitor DAPGreen-positive autophagosomes (green) after SpiA treatment with the indicated doses for 24 h (SpiA) or control untreated cells (Con). The arrows indicate representative cells. (**B**). The DAPGreen (fold) is illustrated in a bar graph (**C**). (**D**) After SpiA treatment for 24 h, Western blotting was performed to assess necroptotic signaling. The total levels of β-actin were used as a loading control for the samples. The relative level (%) normalized to β-actin is illustrated in the bar graph. The red dotted line indicates the boundary marks. * indicates statistical significance at *p* < 0.05. Representative results of three independent experiments.

Since AKT is a key autophagy regulator, we examined autophagic proteins and autophagosomes in human MG63 cells treated with SpiA. Beclin-1 and LC3II levels increased, whereas p62 levels decreased in SpiA-treated cells compared to those in untreated cells, as observed by Western blotting (Figure 4A). Autophagosomes were observed using immunofluorescence. SpiA treatment increased the number of DAPGreen-positive autophagosomes compared to untreated cells (Figure 4B,C). AKT is also an active effector of downstream necroptotic signaling, which is a type of programmed cell death characterized by regulated necrosis. We further evaluated key necroptotic signaling proteins in SpiA-treated human MG63 cells. Western blotting showed a significant decrease in the phosphorylation of receptor-interacting serine/threonine protein kinase (RIP), RIP3, and mixed-lineage kinase domain-like pseudokinase (MLKL) in SpiA-treated cells compared to untreated cells (Figure 4D). These results suggested that SpiA-regulated AKT signaling induces autophagy but blocks necroptosis in human MG63 cells.

### 3.5. SpiA Causes ROS Generation and Mitochondria Potential Loss, and Inhibits the Activation of AKT in Human MG63 Cells

**Figure 5:**
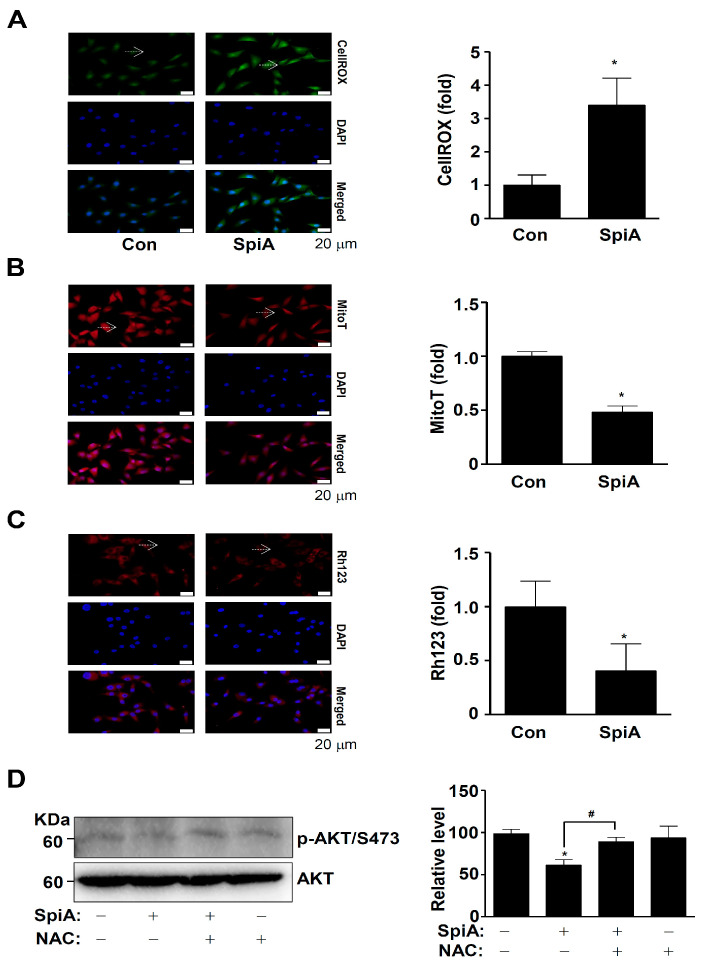
Anti-tumor effects of SpiA on ROS generation and mitochondria potential in human MG63 cells. (**A**,**B**) After treatment with SpiA for 24 h (SpiA) or no treatment (Con), the cells were incubated with CellROX™ Green reagent (**A**) and MitoTracker™ Red CMXRos (**B**) and analyzed under a fluorescence microscope. DAPI staining (blue) indicates nuclei. The arrows indicate representative cells. The relative fold is illustrated in a bar graph. Scale bar: 20 μm. (**C**) Mitochondrial membrane potential was detected using Rhodamine123 (Rh123) under a fluorescence microscope. The arrows indicate representative cells. The Rh123 (fold) is illustrated in a bar graph. Scale bar: 20 μm. (**D**) Cells were treated with SpiA for 24 h in the absence or presence of 5 mM NAC and Western blotting was performed. The total levels of β-actin were used as a loading control for the samples. The relative level (%) normalized to β-actin is illustrated in the bar graph. * and # indicate statistical significance at *p* < 0.05. Representative results of three independent experiments.

ROS is closely associated with the AKT signaling pathway [20]. Thus, we examined whether SpiA was responsible for generating intracellular ROS in human MG63 cells. CellROX™ Green reagent was used to detect levels of intracellular ROS. Treatment with SpiA resulted in an increase in the intensity of the CellROX Green reagent compared to untreated cells (Figure 5A). An ROS increase within cells can cause loss of mitochondrial membrane potential (ΔΨm), leading to mitochondrial dysfunction, cytochrome c release, and apoptosis. We observed mitochondrial membrane potential using MitoTracker™ Red CMXRos and Rh123. As shown in Figure 5B,C, the accumulation of MitoTracker™ Red CMXRos and Rh123 in the mitochondria was significantly reduced after SpiA treatment compared to untreated cells. Next, we investigated the role of ROS in SpiA-induced AKT inactivation. The cells were treated with SpiA with or without pretreatment with the ROS scavenger N-acetyl-l-cysteine (NAC). Western blotting revealed that NAC pretreatment rescued the SpiA-induced decrease in AKT phosphorylation (Figure 5D). These results suggested that SpiA-induced AKT signaling relies on ROS generation and mitochondrial dysfunction to trigger anti-osteosarcoma activity in human MG63 cells.

### 3.6. SpiA Causes Apoptotic Cell Death, Anti-Migration, and Anti-Invasion Effects through ROS Generation in Human MG63 Cells

**Figure 6:**
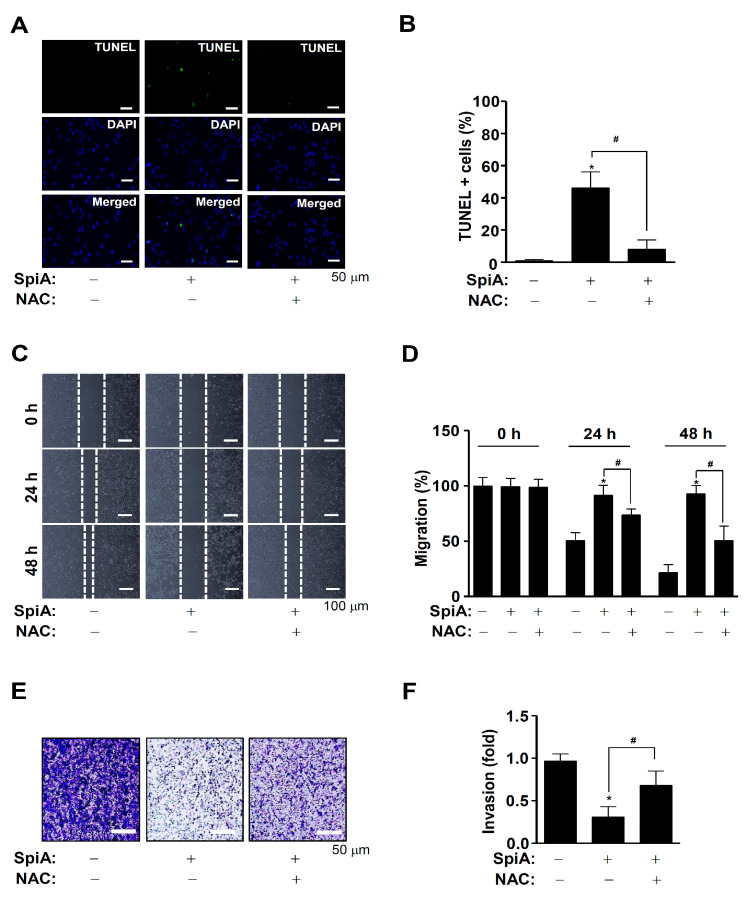
Anti-tumor effects of SpiA-induced ROS on cell death, migration, and invasion in human MG63 cells. (**A**,**B**) Cells were treated with SpiA in the absence or presence of 5 mM NAC for 24 h, and then apoptotic cell death was detected using the TUNEL assay. The relative level (%) is illustrated in the bar graph (**B**). Scale bar: 50 μm. (**C**,**D**) After SpiA treatment for 24 h in the absence or presence of 5 mM NAC, cell migration using the wound assay was monitored under a light microscope. The white dots indicate the movement of cells in the wounded area. (**C**). The migration rate (%) is illustrated in the bar graph (**D**). Scale bar: 100 μm (**E**,**F**). Cell invasion using the Boyden chamber assay was monitored under a light microscope (**E**). The invasion rate (%) is illustrated in the bar graph (**F**). Scale bar: 50 μm. *, and # indicate statistical significance at *p* < 0.05. Representative results of three independent experiments.

We then investigated whether the SpiA-mediated anti-osteosarcoma effects were directly related to ROS generation. Human MG63 cells were treated with SpiA for 24 h with or without NAC pretreatment. The TUNEL assay was used to measure individual apoptotic cell death. Light microscopy indicated that NAC pretreatment prevented SpiA-induced apoptotic cell death in human MG63 cells compared to the control (Figure 6A,B). In addition, the wound-healing assay showed that SpiA decreased cell migration after 24 h compared with the control (Figure 6C,D). However, NAC pretreatment attenuated the anti-migratory effects of SpiA. Finally, we observed cell invasion via extracellular matrix degradation using a Boyden chamber assay. Compared with the control group, SpiA treatment significantly reduced the ability of human MG63 cells to penetrate and migrate through the Matrigel-coated membrane (Figure 6E,F). However, the anti-invasive effects of SpiA were inhibited by NAC pretreatment. These results suggested that SpiA-induced ROS generation triggered anti-osteosarcoma activity in human MG63 cells.

## 4. Discussion

Eight features are common to many tumors and are referred to as “hallmarks of cancer” [36,37,38]. An imbalance between programmed cell death and growth causes benign tumors and normal cells to transform into malignant tumors [37,38]. Natural molecules, which have been used for centuries in traditional medicine, are considered safer and more cost-effective than chemically produced drugs. Consequently, there is growing interest in utilizing natural compounds to develop drugs that can trigger cell death in various cancer types [39,40,41]. Our group has demonstrated the biological activities of natural compounds in human osteosarcoma cells, including apoptosis and metastasis induced through the JAK2/STAT3 pathway after treatment with 4-parvifuran and 4-methyldalbergione isolated from *Dalbergia odorifera*, apoptosis effects from Hederoside C isolated from *Pulsatilla koreana Nakai* through the STAT3 signaling pathway, and autophagy and apoptosis effects from 11-O-Galloyl Bergenin isolated from *Corylopsis coreanas* through AKT inactivation and LC3II upregulation [42,43,44,45]. In the present study, we demonstrated that SpiA, isolated from the dried roots of *L. platyphylla*, induced ROS generation and inhibited the PI3K-AKT-mTOR pathway in human osteosarcoma cells, leading to anti-osteosarcoma effects.

Apoptosis, which mainly results in cell death, is controlled by programmed cellular signaling pathways. These cascades cause cleavage of PARP products, which are the molecular hallmarks of apoptosis. These products lose their ability to repair DNA, resulting in DNA strand break signal loss and cell cycle arrest [46,47,48]. We demonstrated that SpiA promotes cell death by assessing mitochondrial activity via NAD(P)H-dependent cellular oxidoreductase enzymes and found that SpiA induces PARP cleavage in human osteosarcoma cells. We also found that SpiA regulated the expression of several genes associated with apoptosis and cancer progression. SpiA induced the pro-apoptotic Bax protein while reducing the anti-apoptotic Bcl-2 protein, cell cycle proteins (cyclin D1 and Cdk4/6), and proteins related to angiogenesis and metastasis (VEGF and MMP13). Bcl-2 suppresses the translocation of cytochrome c, which blocks the apoptotic process, whereas Bax promotes cytochrome c release [49,50]. Cyclin D1 triggers the G1-to-S phase transition by forming a complex with Cdk4 and Cdk6, which promotes the cell cycle and cell growth in various human malignancies [51,52,53]. Thus, our data suggest that SpiA exerts anti-tumor effects via programmed cell death in human osteosarcoma cells.

We examined the signaling molecules associated with SpiA-induced apoptotic cell death by examining the phosphorylation of intracellular proteins in human osteosarcoma cells. Using the Proteome Profiler Human Phospho-Kinase Array, we found that various proteins were affected by SpiA, particularly AKT, PRAS40, p70S6K, GSK3β, and β-catenin. These proteins are well-known downstream targets of AKT signaling, suggesting that SpiA mainly regulates the AKT-mediated signaling pathway. AKT is a critical signaling protein that regulates multiple key proteins involved in apoptosis, cell cycling, and metastasis during tumorigenesis and progression [54,55]. Notably, the Proteome Profiler Human Phospho-Kinase Array indicated that phosphorylation of AKT (S473) was weaker than that of AKT (T308) under control conditions. Phosphorylation at AKT (T308) in the activation loop and phosphorylation at AKT (S473) in the carboxy terminus are previously reported mechanisms that fully activate AKT [56,57]. Therefore, it was used as a marker of AKT activation. Phosphorylation at Ser473 has been measured and used as an indicator of Akt activity in most studies of Akt in cancer. However, AKT activity in human non-small cell lung cancer is correlated with Akt phosphorylation at Thr308, but not at Ser473 [58]. New phosphorylation events at the carboxyl terminus of AKT (S477 and T479) were recently discovered by Liu et al. [59]. These modifications activate Akt and trigger additional downstream oncogenic cellular activities [59]. These findings suggest that there is a need for in-depth studies on the phosphorylation of AKT in osteosarcoma. Overexpression of AKT has been observed in human osteosarcoma, leading to cell survival, metastasis, and tumorigenesis [60]. Therefore, regulation of AKT-mediated signaling pathways is a critical target for tumor therapy. Consistent with this, SpiA inhibited cell growth and induced apoptosis. Apoptosis interacts with autophagy to prevent tumor growth and metastasis [61,62]. Additionally, in human osteosarcoma cells, the inhibition of AKT signaling leads to autophagy and cell death [63,64]. In the present study, we demonstrated that SpiA promoted autophagy processes through Beclin-1, p62, and the conversion of LC3I to LC3II, and autophagic vacuoles in human osteosarcoma cells. Necroptosis, a type of programmed cell death, also involves AKT signaling [65,66]. SpiA inhibited necroptotic RIP1-RIP3-MLKL proteins in human osteosarcoma cells. It has been demonstrated that AKT inhibitors and apoptotic cascades prevent necroptosis through the necroptotic signaling pathway [67,68]. Similar to our study, SpiA demonstrated anti-proliferative activity against several carcinoma cells and induced autophagy and apoptosis by regulating the PI3K/Akt/mTOR, MAPK, and p53 signaling pathways in human carcinoma HCT116 cells [29]. These findings suggested that SpiA mediates apoptosis, autophagy, and necroptosis by inhibiting the PI3K-AKT-mTOR-p70S6K pathway in human osteosarcoma MG63 cells.

Most anti-cancer treatments rely on ROS-inducing approaches to kill cancer cells by inducing oxidative stress-dependent cytotoxic effects [69,70]. Our results demonstrated that SpiA increased oxidative stress and mitochondrial damage in human osteosarcoma cells. Previous studies have shown that ROS inhibits AKT and its signaling pathway [20,24]. Consistently, we found that the ROS scavenger NAC attenuated SpiA-induced AKT inhibition. It has been reported that ROS can cause cytochrome c release and PARP cleavage through the loss of mitochondrial membrane potential (ΔΨm), eventually resulting in apoptotic cell death [3,71]. Our results also demonstrated that SpiA-induced ROS production led to increased apoptotic cell death. ROS is also associated with cell migration, invasion, and metastasis [72,73,74,75]. Metastatic patients account for approximately 25% of all osteosarcoma cases. However, the 5-year overall survival rate is still less than 30%, and decreased long-term survival is associated with metastatic osteosarcoma [5,72]. In the present study, we found that SpiA suppressed cell migration and invasion through ROS production. AKT signaling regulates metastasis in osteosarcoma cells [76]. SpiA also decreased VEGF and MMP-13 protein levels in human osteosarcoma cells. Metastatic osteosarcoma is caused by the upregulation of MMP-13 [77]. Sauchinone, a lignan isolated from *Saururus chinenesis*, prevents cell migration and invasion by inhibiting Akt-mediated MMP13 expression in human MDA-MB-231 and MTV/TM-011 cells [78]. Thus, our findings suggest that SpiA induces oxidative damage, leading to cell death and anti-metastatic effects in human osteosarcoma cells.

In conclusion, this study is the first to demonstrate that SpiA isolated from the roots of *L. platyphylla* selectively inhibits AKT signaling, blocks cell migration and invasion, and causes apoptotic cell death and autophagy through intracellular ROS generation. Osteosarcoma is a lethal cancer that decreases the patient’s quality of life, and despite recent advancements in treatment, its therapeutic limitations must be overcome. Natural compounds have long been used to treat various diseases, including cancer. Recently, they have gained popularity as potential medication [39,40,41]. Thus, our findings suggest that SpiA is a potential bioactive molecule for use as an anti-osteosarcoma chemotherapeutic drug.

## Figures and Tables

**Table 1:** HPLC Condition.

HPLC Condition	
Column	Phenomenex Kinetex C18 (150 mm × 4.6 mm, 2.6 µm, 100A)
Mobile phase	A (Water with 0.1% Trifluoroacetic acid) B (ACN with 0.1% Trifluoroacetic acid) 2% B (0min) → 100% B (20min) (gradient for 30 min)
Flow rate	1 mL/min
Detector	ELSD
Injection volume	5 µL (1 mg/mL)

## Data Availability

Data are contained with the article.

## Abbreviations

AKT	AKT serine–threonine kinase
*L. platyphylla*	*Liriope platyphylla Wang et Tang*
NAC	N-acetylcysteine
NMR	Nuclear magnetic resonance
MLKL	Mixed lineage kinase domain like pseudokinase
MTT	3-[4,5-dimethylthiazol-2-yl]-2,5-diphenyltetrazolium bromide
mTOR	Mammalian target of rapamycin
p70S6K	70-kDa ribosomal protein S6 kinase
RIP	Receptor-interacting serine/threonine protein kinase
PRAS40	40 kDa proline-rich Akt substrate
SpiA	Spicatoside A
ROS	Reactive oxygen species
TUNEL	Terminal deoxynucleotidyl transferase-mediated FITC–dUDP nick-end labeling
